# Study on the impact of R&D input intensity on technological innovation output ‐ Based on data from China’s high technology industry

**DOI:** 10.1371/journal.pone.0292851

**Published:** 2023-10-17

**Authors:** Chengguo Jin, Dayao Li

**Affiliations:** 1 Beibu Gulf University, Qinzhou, Guangxi, China; 2 Beibu Gulf Marine Development Research Center, Qinzhou, Guangxi, China; East China Normal University, CHINA

## Abstract

With the continuous promotion of China’s innovation-driven development strategy, the role of technological innovation on economic development has become increasingly important. In this context, the support of R&D capital investment for technological innovation also becomes non-negligible. This leads to the question of whether the allocation of R&D capital is reasonable and whether there is room for further improvement. This paper is based on inter-provincial panel data from 2009 to 2020, which are classified based on China’s National Bureau of Statistics for R&D funding sources in high-tech industries and incorporated into an overall discussion framework. Using STATA16 statistical software, the R&D innovation output of high-tech industries is inves-tigated by building a PVAR model with the perspective of funding sources of R&D input intensity. The study results show that (1) the increase in the intensity of enterprises’ own capital investment has a positive impact on innovation output because it can generate a financial "reservoir" effect to support technological innovation. (2) the increase in the intensity of government capital invest-ment has a positive impact on innovation output because it can alleviate the loss of income of en-terprises due to "R&D spillover" and will send a positive signal to the market. (3) the foreign in-vestment intensity has a positive impact on the innovation output of enterprises due to the com-bined effect of "spillover effect" and "crowding out effect". (4) the increase of other capital in-vestment intensity also has a neutral effect on the increase of innovation output under the current financial market environment. Finally, based on the above findings, corresponding policy impli-cations are drawn. This study will help to improve the understanding of R&D capital allocation imbalance and R&D input and output issues in developing countries and provide a reference for policy makers.

## 1. Introduction

Over the past 40 years since China’s reform and opening up, the scale and quality of manufacturing industry have made great leaps, and the economic development has shifted from high-speed growth to high-quality development stage. At this stage, China’s manufacturing industry needs to play the leading role of scientific and technological innovation at a higher level and in a larger scale. As an important main body of technology innovation, the development of high-tech industry is characterized by fast speed, low resource consumption and strong penetration ability to related industries, which is an important power source to promote high-quality economic development. Advanced processes, core technologies, and key production equipment in high-tech industries are playing an increasingly important role in China’s industrial innovation chain at this stage [1, 2]. Continuous investment in R&D is an important guarantee for the industry to achieve high-quality innovation and lasting technological progress. At the same time, the behavioral characteristics of high-tech industries in the new situation are also undergoing profound changes. In order to cope with technological changes and market competition, the R&D investment intensity and investment methods of high-tech industries have a tendency to gradually enhance and expand. Under the wave of the new round of global technological revolution, the imbalance between industrial value creation and capital allocation has become increasingly prominent, and the constraint of R&D capital has become one of the important factors for the lack of industrial innovation power. R&D innovation is characterized by high investment, high risk and long cycle, which is inevitably coupled with effective financial system financing support [3]. Looking at the history of economic development in Europe, America and Japan, we can see that the financial system plays an important role in supporting the technological progress of a country. In this paper, based on the classification of R&D funding sources of high-tech industries in China High Technology Statistical Yearbook and China Science and Technology Statistical Yearbook, R&D funding is divided into four sources: own funds, government funds, foreign funds and other funds, and the R&D investment intensity of each funding source is obtained by adding the data of new product sales revenue of high-tech industries. By incorporating multiple sources of funds into the same analysis framework, a more comprehensive study is conducted that can reflect the overall investment structure of R&D funds in high-tech industries, and the imbalance in the allocation of R&D capital in Chinese high-tech industries is explored from the perspective of R&D investment sources.

The article consists of several sections. Section 1 deals with the introduction. Section 2 is a literature review, which composes the literature from the perspective of R&D funding sources respectively. Section 3 describes the theoretical framework of the study and research hypotheses, which is based on previous studies and presents the research hypothesis of this paper. Section 4 relates to model and data, which introduces the establishment of the PVAR model that incorporates multiple R&D funding sources into the same analysis framework and its data sources. Section 5 presents the empirical test. Section 6 provides a further discussion of the results, analyzing the results of the empirical tests and discussing them with the results of previous studies. Section 7 provides the main findings of the study with policy implications.

## 2. Literature review

### 2.1. On the perspective of own-funded input

Studies on the perspective of own funds input have found that by maintaining product differentiation and product quality improvement, a firm’s R&D activities not only prevent the firm from falling into passive price competition, but also enhance the firm’s efficiency and subsequently its external competitiveness [4, 5]. At the same time, if the market demand corresponding to a firm’s product is greater, the cost reduction effect of R&D activities is more significant, which in turn can also stimulate an increase in R&D activities [6–9]. However, when the economic policy is uncertain, the difficulty of financing will increase and the risk of bankruptcy of the firm will increase, which in turn will cause an increase in the cost of financing, which will eventually drive the level of R&D investment to the level of own funds only [10, 11]. In contrast, under competitive market conditions, only when firms have sufficient funds will they consider bringing in high-technology R&D personnel, acquiring advanced instruments and equipment, and other key factors of production to launch technological R&D activities [12–14].

### 2.2. On the perspective of government-funded input

Studies on the government-funded input perspective have found that the uncertainty of R&D innovation outputs leads to an asymmetry between the deterministic expenditures and risky benefits of R&D activities, which in turn leads to a lack of incentive for firms to innovate technologically [15–17]. Government subsidies, by compensating the corresponding portion of losses incurred by innovative firms due to technology spillovers, can motivate firms to engage in more technological innovation activities, which in turn can enhance the overall technology and welfare of society. When government innovation subsidies are large enough, they also have a catalytic effect on the increase in firms’ own-funded R&D investment [18]. In contrast, the opposite view is that government subsidies have a limited role in promoting firms’ technological innovation, and excessive R&D subsidies may even induce rent-seeking behavior, which in turn inhibits firms’ technological innovation activities [19, 20]. At the same time, the existence of congruent behavior due to government subsidies leads to higher market demand for certain types of R&D innovation resources, which subsequently increases their prices and R&D costs, forcing firms to shift to other investment projects [21]. Therefore, the government may lose social welfare if it blindly subsidizes corporate R&D, so it should target subsidies to companies that are doing well, while it should reduce support to low-end companies [22]. However, the imperfection of the legal system makes it difficult to form a strong constraint on the local government’s subsidy behavior, especially when the subsidy funds are relatively large, the rent-seeking motive of enterprises will become stronger [23]. And the tax credit for corporate R&D has a significant contribution to the R&D innovation activities of firms [24–26].

### 2.3. On the perspective of foreign-funde input

Studies on the perspective of foreign capital investment have found that R&D institutions of multinational companies will have competitive and learning effects on host country companies, prompting host country companies to increase their R&D investment, which in turn increases the technological innovation capacity and the speed of product development in the host country [27, 28]. In the context of economic globalization, the R&D activities of national firms are increasingly internationalized through FDI, which then indirectly or directly enhances the R&D capabilities of the host country [29, 30]. Although the R&D behavior of MNEs may enhance the innovation capacity of the host developing country, it may also have a negative impact on the "high-tech isolated island" of developing countries’ host countries, that is, the innovation achievements cannot be integrated into the economies of developing countries [31–34]. Thus, multinational firms not only dominate foreign direct investment and international trade, but also play an important role in technology diffusion and development [35]. When foreign R&D is embedded in the international production division and R&D system, it enhances the linkages between the host company and upstream and downstream companies at home and abroad, and the lucrative international profits and the availability of important external information increase the motivation and desire of the host company to go global [36]. In turn, multinational firms need to compete with host country firms in order to adapt to the host country’s economic environment, and therefore undertake more R&D activities [37]. In terms of the relationship between foreign institutional inputs and innovation output of Chinese energy firms, there is a significant positive correlation between the two [38]. That is, the rise in a country’s FDI stock has a significant contribution to export technological sophistication [39].

### 2.4. On the perspective of other-funded input

A study on other financial inputs found that A study on other financial inputs found that the higher the level of financial development, the higher the dependence of enterprises on external financing, and the smaller the financing constraints they face, that is, financial development alleviates the financing constraints of enterprises [40, 41]. When the technological innovation of the firm is at the primary stage of tracking and imitation, the innovation risk of the firm can be better controlled and the prospects of the product market are clearer, the capital cost of R&D is relatively low, and the bank-driven financial structure will play an advantage in information collection and processing [42]. The bank route of financing effectively mitigates the problems of adverse selection and moral hazard caused by information asymmetry through collateralization and ex post supervision of the firm [43, 44]. However, the allocation of bank credit funds is more oriented towards policy factors than market factors, resulting in an over-concentration of credit funds in firms with high financing capacity, which may also lead to adverse credit allocation imbalances [45]. Research on equity markets has argued that R&D investment and financing is an important link between financial markets and the market value of firms [46, 47]. The information screening and revealing function in the stock market makes investors pay more attention to the growth of firms [48]. Compared to bank credit financing, securities markets are more able to prospectively direct capital flows to sectors with high growth potential, which in turn drives R&D investment in related areas [49, 50].

In summary, the marginal contributions of this paper lie in the following two aspects: (1) the multiple sources of R&D capital investment are studied within the same analytical framework. In recent years, a large body of literature has focused on the impact of R&D inputs on innovation output, but it has been explored from the perspective of a single source of funding, while a structural and comprehensive study of multiple sources of R&D funding inputs is less common. (2) To address the controversy in the existing literature on whether the impact of each source of R&D inputs on innovation output is facilitative or inhibitory, it is further examined in the overall framework of multiple sources of funding to deepen its understanding in the Chinese context.

## 3. Theoretical analysis and research hypothesis

Enterprises’ R&D innovation activities are expected to improve their production efficiency, deepen their market exploration and strengthen their product competitiveness, and to increase their market share through R&D, and to obtain R&D innovation revenue [5, 6]. According to the "reservoir" effect of enterprises’ financial assets allocation on R&D innovation, when enterprises allocate a higher proportion of their assets in financial assets, it can alleviate the external financing constraints faced by enterprises in R&D innovation, thus forming a financial "reservoir" to support R&D innovation activities. This function of financial "reservoir" supports R&D and innovation activities. This allocation of resources by enterprises can ensure a stable supply of funds for R&D innovation activities, reduce the risk of R&D interruptions, and increase the tolerance for R&D failures, thus improving the output level of R&D innovation [12]. Based on this, research hypothesis H1 is proposed.

**Hypothesis1** H1: The increase in the intensity of proprietary investment has a positive impact on innovation output by creating a financial "reservoir" effect to support R&D innovation.

The fundamental reason for the government to subsidize R&D innovation is the existence of market failure, and the non-exclusive and non-competitive characteristics of innovation results lead to the serious phenomenon of "R&D spillover", i.e., R&D enterprises cannot avoid the benefits of innovation from being captured and exploited by "free-riding" enterprises [15–17]. The uncertainty of R&D innovation and the high Such uncertainty and high risk of R&D innovation weaken enterprises’ willingness to innovate. Therefore, government intervention in the form of R&D subsidies for innovative firms is necessary. R&D subsidies can reduce the uncertainty and riskiness of innovation activities undertaken by firms, while also sending a positive signal to the market that they are recognized by the government and directing external investors to provide more financing [18]. Based on this, research hypothesis H2 is proposed.

**Hypothesis2** H2: An increase in the intensity of government investment can mitigate the loss of revenue due to "R&D spillover" and can send a positive signal to the market, therefore, it has a positive impact on the innovation output of enterprises.

According to the resource dependence theory, innovative enterprises need to continuously introduce high-quality professional talents, high-tech and advanced instruments and high-end equipment, and the addition of foreign institutional investors has a positive contribution to the mobilization of talents and the acquisition of innovative resources. Foreign investors can help innovative enterprises make optimal investment decisions through their own rich investment experience and their ability to collect and analyze information, and at the same time help enterprises build resource platforms at home and abroad to facilitate the transformation of innovation achievements [27, 28]. Based on this, research hypothesis H3 is proposed.

**Hypothesis3** H3: The increase of foreign capital investment intensity positively affects the innovation output of enterprises through the spillover effects of technology spillover, talent spillover and information spillover.

Funding from other sources relying on the financial market can be divided into bank loans, debt and equity financing. Bank loans are used to allocate credit resources through credit intermediaries to achieve the optimal allocation of financial resources, which can help alleviate the problems caused by information asymmetry between the supply and demand of funds, improve corporate governance, form a more stable cooperative relationship with innovative enterprises, and play a positive role in the supply of enterprise R&D funds [42]. Debt and equity financing reduce the cost of information collection between the supply and demand sides and enrich the financing channels of innovative enterprises. Among them, equity financing does not require collateral and repayment, and enables investors to enjoy high returns from corporate innovation and has a natural fit with innovative enterprises [49, 50]. Based on this, research hypothesis H4 is proposed.

**Hypothesis4** H4: The increase in the intensity of other financial inputs enriches the financing channels of innovative enterprises by achieving the optimal allocation of financial resources, and then has a positive impact on the innovation output of enterprises.

## 4. Data source and samples

PVAR model is a multivariate system equation evolved on the basis of VAR model, which has the advantages of three-dimensional variable characteristics and dynamic association equations between multiple correlated variables. It incorporates all exogenous variables and endogenous variables into an endogenous system for analysis, solves the problem of endogeneity caused by the mutual causality between exogenous and endogenous variables, and reduces the time series length requirement of the data. there is a more complex dynamic relationship between R&D input intensity and technological innovation output in multiple channels, and there may be lagged effects. The PVAR model not only takes into account the individual differences among variables, but also incorporates the lag effect into the system for analysis, which can portray the dynamic process of the impact path of R&D input intensity on technological innovation in a multi-level and multi-angle manner. Based on this, the PVAR model is constructed as follows:

$$y_{it}\;=\;\alpha_{0}\;+\;\sum_{j=1}^{n}A_{j}y_{it-1}\;+\;f_{i}\;+\;d_{i}\;+\;\varepsilon_{it}$$ (1)

In Eq (1), α_0_ represent the intercept term, A_j_ represent the estimated coefficient matrix, y_it_ is a vector group composed of five endogenous variables, y_it-j_ represent the lagged vector group, i represent the ith province (municipality directly under the central government and autonomous region), t is the t_th_ year, j represent the lagged order, f_i_, d_i_ and ε_it_ is the fixed effect, time effect and random disturbance terms, respectively.

The expanded equation is as follows.


$$\left[\begin{array}{l} La_{it}\\ Sf_{it}\\ Gf_{it}\\ Ff_{it}\\ Of_{it} \end{array}\right]=\alpha_{0}+\sum_{j=1}^{n}\left[\begin{array}{l} La_{it-j}\\ Sf_{it-j}\\ Gf_{it-j}\\ Ff_{it-j}\\ Of_{it-j} \end{array}\right]+f_{i}+d_{i}$$ (2)

In Eq (2), La is innovation output, which is measured by the number of patents in high-tech industries; Sf is the intensity of own capital investment, which is measured by the ratio of R&D funds from corporate sources to new product sales revenue; Gf is the intensity of government capital investment, which is measured by the ratio of R&D funds from government sources to new product sales revenue; Ff is the intensity of foreign capital investment, which is measured by the ratio of R&D funds from foreign sources to new product sales revenue; Of is the intensity of other capital investment, which is measured by the ratio of R&D funds from other sources to new product sales revenue. Ff is the investment intensity of foreign capital, which is the ratio of R&D funds from foreign capital to new product sales revenue; Of is the investment intensity of other capital, which is the ratio of R&D funds from other sources to new product sales revenue. The data are obtained from the China High Technology Industry Statistical Yearbook, China Industrial Statistical Yearbook and China Science and Technology Statistical Yearbook. In order to facilitate the study, the sample was processed as follows: (1) The sample was subdivided into R&D investment from own funds, R&D investment from government funds, R&D investment from foreign funds and R&D investment from other funds, and incorporated into the value of new product sales revenue respectively, and the intensity value of R&D investment from each channel was taken. (2) The four provinces of Tibet, Hainan, Ningxia and Qinghai, which had more missing data, were excluded. The remaining part of the missing data is processed by mean interpolation method.

The results of descriptive statistics of the variables are shown in Table 1.

**Table 1:** Descriptive statistics of variables.

Variable	Mean	Std. Dev.	Min	Max
la	3675.10	8466.66	502.00	74083.00
sf	1.27307	7.06795	0.000551	123.4665
gf	0.47369	2.67786	0.00405	47.34931
ff	0.01061	0.07510	0.00007	1.29383
of	0.05722	0.24393	0.00092	4.22814

## 5. Empirical test

### 5.1. Descriptive statistical analysis

The PVAR model requires the variables to be stationary, otherwise it may produce the problem of "pseudo-regression" and affect the scientificity of the estimation results. Therefore, a panel unit root test of the five variables is required to determine the smoothness of the variables. Based on the applicability principle, the LLC and HT tests were chosen in this paper, and the results are shown in **Table 2**, and all the variables have passed the smoothness test.

**Table 2:** Unit follow test.

Variables	Symbol	LLC Inspection	Prob	HT Inspection	Prob	Result
Innovation output	La	-7.0733	0.0000	0.7812	0.0000	smooth
own-funded intensity	Sf	-3.0896	0.0081	0.8208	0.0000	smooth
government-funded intensity	Gf	-6.2549	1.0000	0.7533	0.0000	smooth
foreign-funded intensity	Ff	-5.4704	0.0000	0.8184	0.0000	smooth
other-funded intensity	Of	-2.0655	0.0743	0.7715	0.0000	smooth

### 5.2. Lag order

To ensure the validity of the PVAR model parameter estimation results, the optimal lag order of the model needs to be selected. Choosing too small a lag order will result in serious loss of sample data; choosing too large will reduce the degrees of freedom of the model parameters. Therefore, the determination of the optimal lag order of the PVAR model is crucial. In this paper, the optimal lag order is selected based on the AIC, BIC and HQIC Guidelines, and the results are shown in Table 3, and each criterion is shown to support lag order 2 as optimal.

**Table 3:** Hysteresis order.

Lag	AIC Guidelines	BIC Guidelines	HQIC Guidelines
1	17.758	19.074	18.723
2	17.4103*	18.810*	18.185*
3	18.652	20.820	19.735
4	19.004	22.246	20.911
5	20.011	22.196	20.595
6	19.513	20.573	18.687

### 5.3. Analysis of enterprise characteristic variables

The Granger causality test allows the analysis of the dynamic relationship between the variables and the determination of the rationality of the current variables to be included in the same PVAR system. The results of the Granger test are shown in Table 4. The increase of own capital input intensity Sf and government capital input intensity Gf are Granger causes of the increase of innovation output La at 1% significant level, while the increase of foreign capital input intensity Ff and other capital input intensity Of are not Granger causes of the increase of innovation output La. None of the increase in innovation output La is the Granger cause of the increase in each of the other variables, indicating a strong policy bias and the need for further improvement in marketization. And the increase of each variable is the Granger cause of the increase of other variables, with mutual promotion effect, indicating a stronger bunching effect of funds, which may be more concentrated in key areas.

**Table 4:** Granger causality test.

Symbol	Cause and Effect	Chi2	Result
La	Sf is not Granger reason	16.602	Reject***
	Gf is not Granger reason	22.915	Accept
	Ff is not Granger reason	0.560	Accept
	Of is not Granger reason	1.734	Accept
Sf	La is not Granger reason	0.622	Accept
	Gf is not Granger reason	14.224	Reject***
	Ff is not Granger reason	8.367	Reject *
	Of is not Granger reason	10.079	Reject**
Gf	La is not Granger reason	1.558	Accept
	Sf is not Granger reason	9.110	Reject**
	Ff is not Granger reason	11.613	Reject**
	Of is not Granger reason	13.736	Reject***
Ff	La is not Granger reason	1.855	Accept
	Sf is not Granger reason	9.348	Reject**
	Gf is not Granger reason	14.711	Reject***
	Of is not Granger reason	9.599	Reject**
Of	La is not Granger reason	0.817	Accept
	Sf is not Granger reason	10.774	Reject**
	Gf is not Granger reason	7.936	Reject*
	Ff is not Granger reason	9.416	Reject**

### 5.4. Panel granger causality

Since the PVAR model is a dynamic model with more complex interactions among variables, it is difficult to accurately determine the impact of a change in one variable on other variables. the impulse response function of the PVAR model can describe the impact of the orthogonalized information of a variable within the model on each variable in the model, and analyze the dynamic response of each variable to a unit shock generated by the current and future the impact of each shock variable on the other variables.

**Fig 1:**
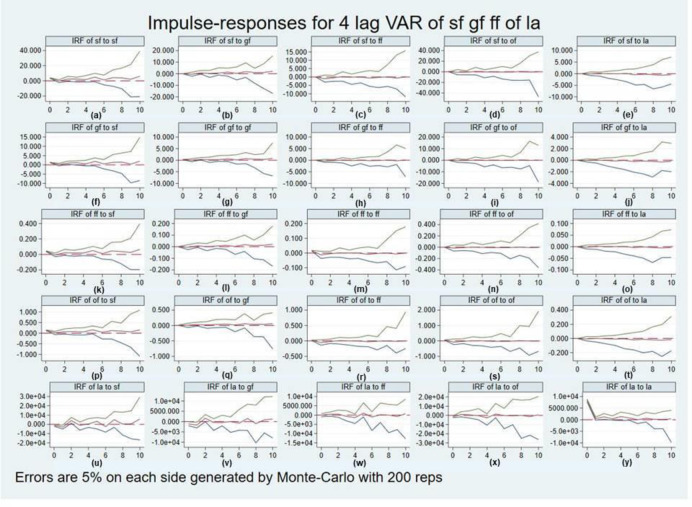
Panel granger causality.

The result is shown in Fig 1, which contains 5 rows and 5 columns with a total of 25 figures. Fig 1(A)–1(E) in the first row show the dynamic responses of own funds intensity (Sf) after a one-unit shock to each variable. Fig 1(F)–1(J) in the second row show the dynamic response of government funding intensity (Gf) after a one-unit shock to each variable. Fig 1(K)–1(O) in the third row show the dynamic response of foreign financial intensity (Ff) after a one-unit shock to each variable. Fig 1(P)–1(T) in the fourth row show the dynamic response of other financial intensity (Of) after a one-unit shock to each variable. Fig 1(U)–1(Y) in the fifth row show the dynamic response of innovation output (La) after a one-unit shock to each variable.

Fig 1(A), 1(F), 1(K), 1(P) and 1(U) in the first column indicate the dynamic responses of each variable after a one-unit shock to each variable from the intensity of own funds (Sf), respectively. Fig 1(B), 1(G), 1(L), 1(Q) and 1(V) in the second column indicate the dynamic responses of each variable after a one-unit shock to each variable from government funding intensity (Gf), respectively. Fig 1(C), 1(H), 1(M), 1(R) and 1(W) in the third column indicate the dynamic responses of each variable after a one-unit shock to each variable from foreign funding intensity (Ff), respectively. Fig 1(D), 1(I), 1(N), 1(S) and 1(X) in the fourth column indicate the dynamic responses of each variable after a one-unit shock to each variable from other financial intensity (Of), respectively. Fig 1(E), 1(J), 1(O), 1(T) and 1(Y) in the fifth column indicate the dynamic responses of each variable after a one-unit shock to each variable from innovation output (La), respectively.

In this paper, we focus on the impulse responses of the fifth row Fig 1(U)–1(Y) and analyze them.

First, observing the Fig 1(U), it can be seen that when the innovation output (La) is subjected to a shock of one unit of own funds intensity (Sf), two negative troughs appear in periods 1 and 6, respectively, during the 10 period, with the minimum value appearing in period 1. Three positive peaks occur in periods 2, 5 and 7, with the maximum value occurring in period 7. Overall, the increase in the intensity of own funds has a positive contribution to the increase in innovation output of high-tech industries over time from negative to positive.

Second, observing Fig 1(V), it can be seen from the figure that when innovation output (La) is shocked by one unit of government funding intensity (Gf), two negative troughs appear in periods 6 and 8, respectively, during the 10 period, with the minimum value appearing in period 8. Two positive peaks occur in periods 2 and 7, with the maximum occurring in period 2. Overall, the increase of government funding intensity on the increase of innovation output of high-tech industries shows a positive boost from negative to positive over time.

Third, observing Fig 1(W), it can be seen from the figure that when innovation output (La) is shocked by one unit of foreign funding intensity (Ff), three negative troughs appear in periods 4, 6, and 9, respectively, during the 10 period, with the minimum value appearing in period 4. Two positive peaks occur in periods 2 and 5, with the maximum occurring in period 5. Overall, the increase in foreign capital intensity shows a neutral effect on the increase in innovation output of high-tech industries over time.

Fourth, observing Fig 1(X), it can be seen that when innovation output (La) is shocked by one unit of other financial intensity (Of), two negative troughs appear in periods 4 and 9, respectively, during the 10 period, with the minimum value appearing in period 4. A positive crest and a maximum value occurs in period 5. Overall, the increase in the intensity of other funds shows a neutral effect on the increase in innovation output of high-tech industries over time.

Fifth, it can be concluded from the Fig 1(Y) that when the innovation output (La) is subjected to one unit of own shock, the maximum positive shock is generated in the current period within 10 periods, and then gradually decreases and tends to zero from period 8. It shows that the innovation output capacity of high-tech industries is affected by the larger prior innovation output capacity, showing a strong cumulative effect.

### 5.5. Variance decomposition

The impulse response function of the PVAR model can reflect the dynamic effects among the variables, while the further variance decomposition can resolve the variance of the variables to the individual disturbance terms and determine the degree of contribution of the shocks to the coefficient of variation of the errors of the endogenous variables.

As shown in Table 5, the contribution of innovation output (La) to its own error coefficient of variation decreases from 0.935 in period 1 to 0.422 in period 10. the contribution of own funds intensity (Sf) to the error coefficient of variation of innovation output (La) increases from 0.016 in period 1 to 0.398 in period 10. the contribution of government funds intensity (Gf) to the error coefficient of variation of innovation output (La) increases from 0.018 in period 1 to 0.061 in period 4, then decreases to 0.052 in period 6 and increases again to 0.065 in period 10. The contribution of the coefficient of variation of government funding intensity (Gf) to innovation output (La) increases from 0.018 in period 1 to 0.061 in period 4, then decreases to 0.052 in period 6, then increases again to 0.065 in period 10. The contribution of the coefficient of variation of the error of foreign funding intensity (Ff) to innovation output (La) increases from 0.000 in period 1 to 0.033 in period 8, then decreases to 0.028, then increases again to 0.030. The coefficient of error variation of other financial intensity (Of) on innovation output rises from 0.031 in period 1 to 0.093 in period 6, then decreases to 0.075 in period 9 before rising again to 0.084 in period 10.

**Table 5:** Variance decomposition.

Response Variable	Period number	Shock variable				
		La	Sf	Gf	Ff	Of
La	1	0.935	0.016	0.018	0.000	0.031
	2	0.794	0.119	0.047	0.002	0.039
	3	0.655	0.246	0.061	0.004	0.034
	4	0.645	0.251	0.061	0.008	0.035
	5	0.603	0.230	0.058	0.022	0.088
	6	0.543	0.285	0.052	0.027	0.093
	7	0.514	0.311	0.053	0.033	0.088
	8	0.432	0.406	0.058	0.028	0.076
	9	0.430	0.404	0.063	0.028	0.075
	10	0.422	0.398	0.065	0.030	0.084

As mentioned above, in the context of Chinese high-tech industries, the intensity of own-funding input has a positive contribution to innovation output [3, 5–9]. Similarly, the intensity of government funding input also has a positive contribution to innovation output [18]. In other words, under the overall framework of multiple R&D funding sources, the above two sources of R&D funding now play an important role in innovation output, and the high-tech industry has entered a new stage of mainly independent innovation compared with the past model of technology introduction and imitation [24–26]. The intensity of foreign capital investment, on the other hand, shows a neutral impact [31–33], which still holds in the overall framework of multiple sources of R&D funding. The intensity of other capital investment also shows a neutral impact, which supports the studies of Kou et al., Li et al. and Harrison et al. [49, 50]. That is, the above two sources of R&D funding have room for further adjustment and improvement in the structure of R&D funding in high-tech industries.

## 6. Result and discussion

According to the analysis of Granger causality test, impulse response and variance decomposition in the previous section, it is clear that. First, the increase in the intensity of own capital investment has a positive and positive contribution to the increase in innovation output. The possible reasons for this are that, on the one hand, in the general environment of global industrial chain reshaping and China’s industrial restructuring, enterprises’ technological innovation is usually market demand-oriented in order to gain competitive advantages at home and abroad for higher profits, and their own funds R&D investment is more targeted to meet the needs of different markets, and the innovation output is more likely to realize the transformation of results. In addition, the use of private funds is less subject to external pressure and restrictions, so that enterprises can optimize the allocation of innovation resources more effectively and improve the efficiency of technological innovation. On the other hand, the level of R&D investment of own funds conveys to a certain extent the level of innovation output and capability of enterprises to the outside world, which in turn helps enterprises to obtain further external financial support and R&D cooperation opportunities.

Second, the Chinese government has long adopted a series of financial subsidies and tax concessions to guide and stimulate enterprises’ technological innovation; therefore, consistent with expectations, the increase in the intensity of government funding investment shows a positive contribution to the increase in innovation output, which may be attributed to the following reasons: on the one hand, government R&D funding can compensate for enterprises’ shortage in R&D funding, reduce their R&D costs On the one hand, government R&D funds can compensate for the shortage of R&D funds of enterprises, reduce their R&D costs and risks, and alleviate the technology spillover problem of unequal private investment and social returns, which in turn contributes to the healthy development of the overall R&D activities of the industry. On the other hand, the government’s guidance of collaboration among different R&D innovation subjects can promote the optimal allocation of social R&D resources, such as "collaborative innovation fund" and "industry-university-research innovation fund", which promote the synergy effect of R&D activities and improve the efficiency of technological innovation. The synergistic effect of R&D activities is promoted and the efficiency of technological innovation is improved.

Again, with the deepening of global economic integration, the technological exchange and cooperation between Chinese domestic enterprises and foreign enterprises are getting closer and closer. According to the results of the previous empirical analysis, the increase in the intensity of foreign capital investment at this stage shows a neutral effect on the increase of innovation output, the possible reason for this is that the positive effect of foreign capital investment intensity on the technological innovation output of China’s high-tech industries is offset by the negative effects of reverse technology diffusion, monopoly and dependence. The purpose of foreign-invested enterprises and institutions to conduct R&D activities in the host country is to obtain economic profit, and the intensity of foreign capital investment brings technology spillover effect to Chinese enterprises while also suppressing the efficiency of technological innovation output of Chinese enterprises to a certain extent, generating a crowding-out effect. While the occurrence of spillover effect and crowding-out effect will change with the change of environment, at this stage, the effect of increasing the intensity of foreign capital investment on the increase of innovation output of Chinese high-tech industries is not obvious.

Finally, the increase in the intensity of other financial inputs also shows a neutral effect on the increase of innovation output. The financial market environment, as the external financing environment for enterprises, can alleviate the financing constraints in R&D investment activities, but the mismatch of financial factors is still widespread in China, leading to the problems of restricted capital circulation and inefficient capital allocation. In addition, the current financing mode of China’s financial market is still dominated by the banking system of indirect financing. Banks are relatively easier to alleviate information asymmetry in the production mechanism and have advantages in supervision, but it is not easy for startups and private enterprises to obtain credit support and the financing cost is relatively high. The issuance of stocks and bonds in the capital market can alleviate information asymmetry and "free-rider" problems, but the post-facto supervision is relatively insufficient and the issuance threshold is high. Therefore, the increase in the intensity of other financial inputs does not have a significant impact on the increase in innovation output of Chinese high-tech industries.

## 7. Conclusion and policy recommendations

### 7.1. Conclusions

This paper takes the high-tech industries in 28 provinces of China from 2009 to 2020 as the research object, measures the technological innovation output by the number of patent applications, and classifies the innovation capital investment intensity into own capital investment intensity, government capital investment intensity, foreign capital investment intensity and other capital investment intensity according to the source channels. The PVAR model was used to empirically test the impact of R&D investment intensity of each channel on technological innovation output, and the following conclusions were drawn: (1) The increase in the intensity of enterprises’ own capital investment and government capital investment has a positive effect on the increase of innovation output. The hypothesis of this paper is supported by the hypothesis H1: the increase in the intensity of private capital investment has a positive impact on innovation output by generating a financial "reservoir" effect to support R&D innovation; H2: the increase in the intensity of government capital investment can mitigate the loss of revenue of enterprises due to "R&D spillover". H2: The increase of government investment can alleviate the loss of income of enterprises due to "R&D spillover", and can send positive signals to the market, therefore, it has a positive impact on the innovation output of enterprises. (2) The increase in the intensity of foreign capital investment and other capital investment shows a neutral impact on the increase of innovation output at this stage. The hypothesis H3 of this paper is not supported: the increase in the intensity of foreign capital investment has a positive impact on the innovation output of enterprises through the spillover effects of technology spillover, talent spillover and information spillover; H4: the increase in the intensity of other capital investment has a positive impact on the innovation output of enterprises through the optimal allocation of financial resources and the enrichment of financing channels for innovative enterprises.

### 7.2. Policy recommendations

In response to the above findings, the following research implications are proposed.

First, in the R&D strategy decision of enterprises, it is encouraged to reasonably set the R&D investment intensity, ensure the stability and continuity of R&D fund supply, optimize the structure of R&D fund use according to enterprises’ own type, and improve the efficiency of fund combination. To actively break the "curse" of resources in the process of independent innovation, further strengthen the cooperation among enterprises, enterprises, universities and research institutions, etc., make timely and continuous forecasts on the stage of technology life cycle and future evolution direction in the industry, provide a basis for scientific decision-making, strengthen core technology research and reduce technological dependence.

Second, further improve the government’s science and technology innovation support policies at all levels, determine the intensity of government capital investment according to the spillover of R&D innovation, base on the guiding position of government funds, and reserve space for social capital investment. Improve the decision-making and operation mechanism of government fund investment, improve the supervision mechanism of fund deployment and use and evaluation mechanism of use effectiveness, and form a systematic and perfect supervision system of fund use. Improve the efficiency of the use of government funds. Coordinate and promote the ecosystem construction of industrial innovation clusters.

Third, through a variety of measures to increase the opening of foreign enterprises, At the same time to improve the negative list mechanism, and to promote the optimal allocation of various factors. Reduce excessive intervention and strengthen the supervision mechanism. Make full use of foreign capital investment in core technologies to increase research efforts. Take the innovation-driven development strategy as the guide, strengthen regulations to protect against disorderly expansion of capital and avoid capital overstepping.

Fourth, continue to deepen the financial supply-side structural reform, unblock the conduction channels, and enhance the effectiveness of the role of financial deepening to promote industrial technology innovation. Smooth the credit conduction channels, enhance the availability of credit funds for innovative enterprises, and improve the construction of credit guarantee system. Reasonably lower the threshold of access to the capital market, reduce the market power of head financial institutions, lower the financing cost of innovative enterprises, and promote the high-quality development of supply chain finance.

### 7.3. Limitations

The research limitations and research outlook of this paper are: (1) This paper takes China as a whole as the research object, ignoring the differences in technology development levels and industrial development between regions in eastern, central and western China, which will be further studied in depth in the future. (2) This paper does not subdivide the different types of enterprises in high-tech industries, and the study will be further enriched and improved in the future.

## Supporting information

S1 Data Supporting data for the study. (ZIP)
